# Puerarin affects adipose lipolysis and ameliorates obesity by gut microbiota

**DOI:** 10.3389/fmicb.2025.1567339

**Published:** 2025-04-03

**Authors:** Zhaoyi Li, Yingyan Ye, Shanglei Lai, Jianfeng Bao, Ai Fu

**Affiliations:** ^1^Institute of Hepatology and Epidemiology, Affiliated Hangzhou Xixi Hospital, Zhejiang Chinese Medical University, Hangzhou, China; ^2^Hangzhou Linan District People's Hospital, Hangzhou, China; ^3^Department of Medical Research Center, Shaoxing People's Hospital, Shaoxing, China; ^4^Department of Hepatology, Affiliated Hangzhou Xixi Hospital, Zhejiang Chinese Medical University, Hangzhou, China

**Keywords:** puerarin, obesity, gut microbiota, adipose, lipolysis

## Abstract

**Background:**

Obesity has become a widespread metabolic disorder, marked by its escalating global prevalence. Puerarin, extracted from *Pueraria lobata*, one of the traditional homologies of medicine and food, demonstrates anti-obesity properties. Nonetheless, the mechanisms through which puerarin exerts its anti-obesity effects remain to be elucidated. This study seeks to highlight the potential application of puerarin in obesity management and to investigate the underlying mechanisms involving gut microbiota and lipid metabolism.

**Method:**

Different doses of puerarin were administered to the high-fat diet mice model, and the structure and composition of gut microbiota were analyzed using 16S rRNA sequencing. Q-PCR evaluated the expression of genes related to lipid metabolism.

**Result:**

Our findings demonstrate that puerarin treatment significantly reduces body weight and epithelial and beige fat mass. Furthermore, puerarin alters the structure and composition of gut microbiota, which is associated with metabolism. Additionally, puerarin treatment significantly upregulates gene expression, fatty acid transport protein 5 (FATP5) hormone-sensitive lipase (HSL), adipose triglyceride lipase (ATGL) in epithelial and beige adipose.

**Conclusion:**

Puerarin demonstrates potential in ameliorating obesity by changing the structure and composition of gut microbiota, which enhances the transport of fatty acid and triglycerides hydrolysis within adipose tissue. This study provides a novel perspective on puerarin as a dietary supplement for obesity.

## 1 Introduction

Obesity, due to energy accumulation excess associated with metabolism disorders, constitutes a global epidemic, with its prevalence persistently rising. Over 2 billion, nearly 30% of the world population individuals are impacted by obesity (Caballero, 2019). Obesity is considered a long-term management chronic disease with mild or severe metabolism disorders and long-term low inflammation, which will increase the risk of many physiological problems such as non-alcoholic fatty liver disease, type 2 diabetes, and cancers.

Some evidence indicates that microbiota, especially gut microbiota, represents a significant environmental factor influencing the progression of obesity (Virtue et al., 2019). The human gut houses roughly 100 trillion microorganisms (Gomes et al., 2018). Variations in the composition and structure of the microbiota can affect body weight. For example, some studies suggest that a decrease in Bacteroides and an increase in Firmicutes may contribute to obesity (Walters et al., 2014). Moreover, diet and lifestyle intricately interact with gut microbiota and metabolites, exerting beneficial and detrimental effects. These changes in gut microbiota and their byproducts can influence weight by impacting short-chain fatty acids, fat metabolism, and appetite (Cuevas-Sierra et al., 2019). Utilizing dietary interventions or nutritional supplements to manage obesity through the modulation of gut microbiota represents a promising approach.

*Pueraria lobata* is a Chinese traditional prevalent and extensively utilized medicinal and edible plant. It is rich in various compounds, including isoflavones, flavones, coumarins, and sterols, which showcase remarkable therapeutic potential. These phytochemicals contribute to *Pueraria lobata*’s diverse pharmacological properties, particularly its antioxidant, hepatoprotective, antidiabetic, and cardiovascular protective effects (Wang et al., 2020). Puerarin is a kind of isoflavone and is one of its efficacious components derived from the roots (Wang et al., 2023). This compound is frequently employed as a dietary supplement (Zhou et al., 2014). Puerarin demonstrates several beneficial pharmacological effects, including the mitigation of liver damage caused by metabolic factors through the reduction of lipid accumulation and alleviation of liver inflammation (Dong et al., 2024). This suggests that puerarin may improve lipid metabolism disorders. However, further research is needed to understand how puerarin ameliorates obesity. Additionally, studies indicate that puerarin can alleviate fat absorption in the gut through the brain-to-gut axis in rats by the dorsal motor nucleus of the vagus targeting jejunum microvilli (Lyu et al., 2024). Nevertheless, the exact mechanism by which puerarin addresses obesity through gut microbiota remains unclear.

In this study, we aimed to explore the mechanism of pueraria affecting lipid metabolism through gut microbiota. We compared the changes in body weight and blood biochemical levels of high-fat-fed mice treated with puerarin and analyzed their microbiota using 16r sequencing technology. Additionally, we explored the impact of puerarin on adipose tissue synthesis in mice to elucidate its anti-obesity mechanism. Our results propose a novel approach to combating obesity by utilizing puerarin to modify the gut microbiota composition and enhance adipose tissue breakdown in mice. This research helps us understand the role of puerarin in treating obesity.

## 2 Materials and methods

### 2.1 Animals and treatment

The male C57BL/6 mice were purchased from Hangzhou Qizhen Experimental Animal Technology (Hangzhou, China) and housed in the Animal Experimental Centre of Zhejiang Traditional Chinese Medicine University. The animal experiments were approved by the Ethics Committee of Zhejiang Chinese Medical University (I ACUC-20220815-15). After a week of adaptive feeding, the mice were randomly divided into the control group (NFD), which was fed a standard diet; the high-fat group (HFD), which was fed a 60% high-fat diet; according to described previously (Li et al., 2024), the puerarin (HPLC ≥ 98%) (Solarbio, Beijing, China) treatment groups, which was fed a 60% high-fat diet and gavaged every day by 200 mg/kg or 300 mg/kg, respectively, (HFD200 and HFD300) (Research Diets, New Brunswick, United States). The body weight of mice was recorded every week. The plasma and adipose tissues were collected after mice were sacrificed and stored at −80°C.

### 2.2 Biochemistry

The biochemical analyzer detected the levels of plasma triglyceride (TG), high-density lipoprotein (HDL), and glucose (GLU).

### 2.3 Histological analysis

The epithelial adipose tissue was fixed in the 4% paraformaldehyde solution, embedded in paraffin, and the wax block was cut to a 5–6 μm thickness. Hematoxylin and eosin (H&E) staining was applied to assess the size of lipid droplets.

### 2.4 16S rRNA gene sequencing analysis

The DNA was extracted from the feces of mice. Hangzhou Mingke Biotechnology performed 16S rRNA gene sequencing. We performed OTU clustering analysis and species taxonomy analyses based on the results obtained, finding different microbial communities. Using the PICRUSt R package to predict the function of microorganisms.

### 2.5 Quantitative real-time PCR

Total RNA was extracted from mice tissues and cultured cells by Trizol kit (Invitrogen, NY, United States). After quantitating the extracted RNA, synthesize the cDNA as described in the manual by HiScript III First Strand cDNA Synthesis Kit (Vazyme, Nanjing, China). QRT-PCR was performed with cDNA, primer, and SYBP Green by using CFX Opus 384 real-time PCR system (Bio-rad Laboratories Ltd., CA, United States). The primer sequence is as follows (Table 1).

**Table 1:** List of primer sequences.

Gene	Forward primer	Reverse primer
*Acc1*	AGGTACAGTAAGAGCCATAGGAC	CTTGGTTGTCAAAATGCCATCAG
*Scd1*	TTCTTGCGATACACTCTGGTGC	CGGGATTGAATGTTCTTGTCGT
*Fasn*	TAT CCT GCT GTC CAA CCT CAG CAA	TCA CGA GGT CAT GCT TTA GCA CCT
*Hsl*	TCCCTCAGTATCTAGGCCAGA	GGCTCATTTGGGAGACTTTGTTT
*Atgl*	CAACGCCACTCACATCTACGG	GGACACCTCAATAATGTTGGCAC
*Cd36*	ATGGGCTGTGATCGGAACTG	GTCTTCCCAATAAGCATGTCTCC
*Dgat2*	AGTGGCAATGCTATCATCATCGT	TCTTCTGGACCCATCGGCCCCAGGA
*Fatp5*	CTACGCTGGCTGCATATAGATG	CCACAAAGGTCTCTGGAGGAT

### 2.6 Statistical analyses

We used mean ± SD to present the data and GraphPad Prism to analyse the significant differences. *T*-test and two-way ANOVA were used to analyse the statistical significance. The analysis of gut microbiota data was completed on the Majorbio platform.^1^ *p* < 0.05 means a significant difference.

## 3 Results

### 3.1 Puerarin alleviates high-fat diet-induced hyperlipidemia and obesity

**Figure 1:**
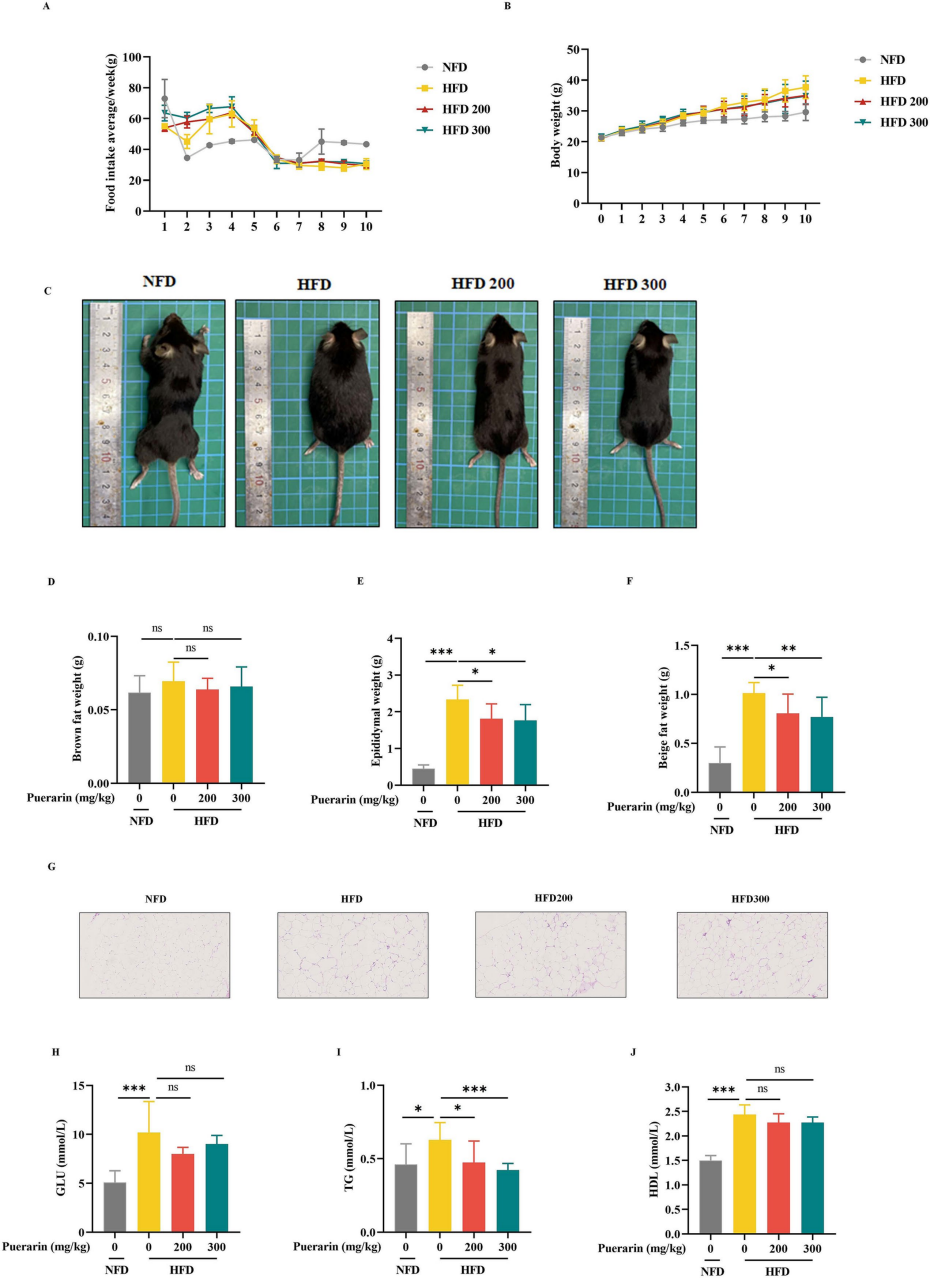
Puerarin alleviates high-fat diet-induced hyperlipidemia and obesity. **(A)** Mice food intake; **(B)** Body weight; **(C)** Representative images of mice; **(D)** Brown weight; **(E)** Epididymal fat weight; **(F)** Beige fat weight; **(G)** The H&E analysis of Epididymal fat; **(H)** The plasma GLU levels; **(I)** The plasma TG levels; **(J)** The plasma HDL. NFD, normal fat diet; HFD, high-fat diet; HFD200, mice were treated with 200 mg/kg/day puerarin with a high-fat diet; HFD 300, mice were treated with 300 mg/kg/day puerarin with a high-fat diet. *means *p* < 0.05, **means *p* < 0.01, ***means *p* < 0.001. The following markings have the same meaning.

To investigate the effect of puerarin on obesity and hyperlipidemia, the mice were treated with different doses of puerarin for 6 weeks after 4 weeks of a high diet. Although puerarin treatment had no difference in food consumption (Figure 1A), puerarin significantly decreased the body weight of mice (Figures 1B,C). Compared with HFD group, puerarin treatment substantially decreases the weight of epididymal fat and beige fat (Figures 1E,F). Moreover, epididymal adipose histological analysis revealed puerarin intervention significantly reduced the size of lipid droplets in epididymal fat (Figure 1G). However, there is no difference between the HFD group and the puerarin treatment group (Figure 1D). Compared with the HFD group, puerarin (200, 300 mg/kg) significantly reduced plasma TG (Figure 1I). However, we did not observe puerarin treatment inhibit plasma HDL and GLU (Figures 1H,J).

### 3.2 Puerarin intervention alters the structure of gut microbiota

**Figure 2:**
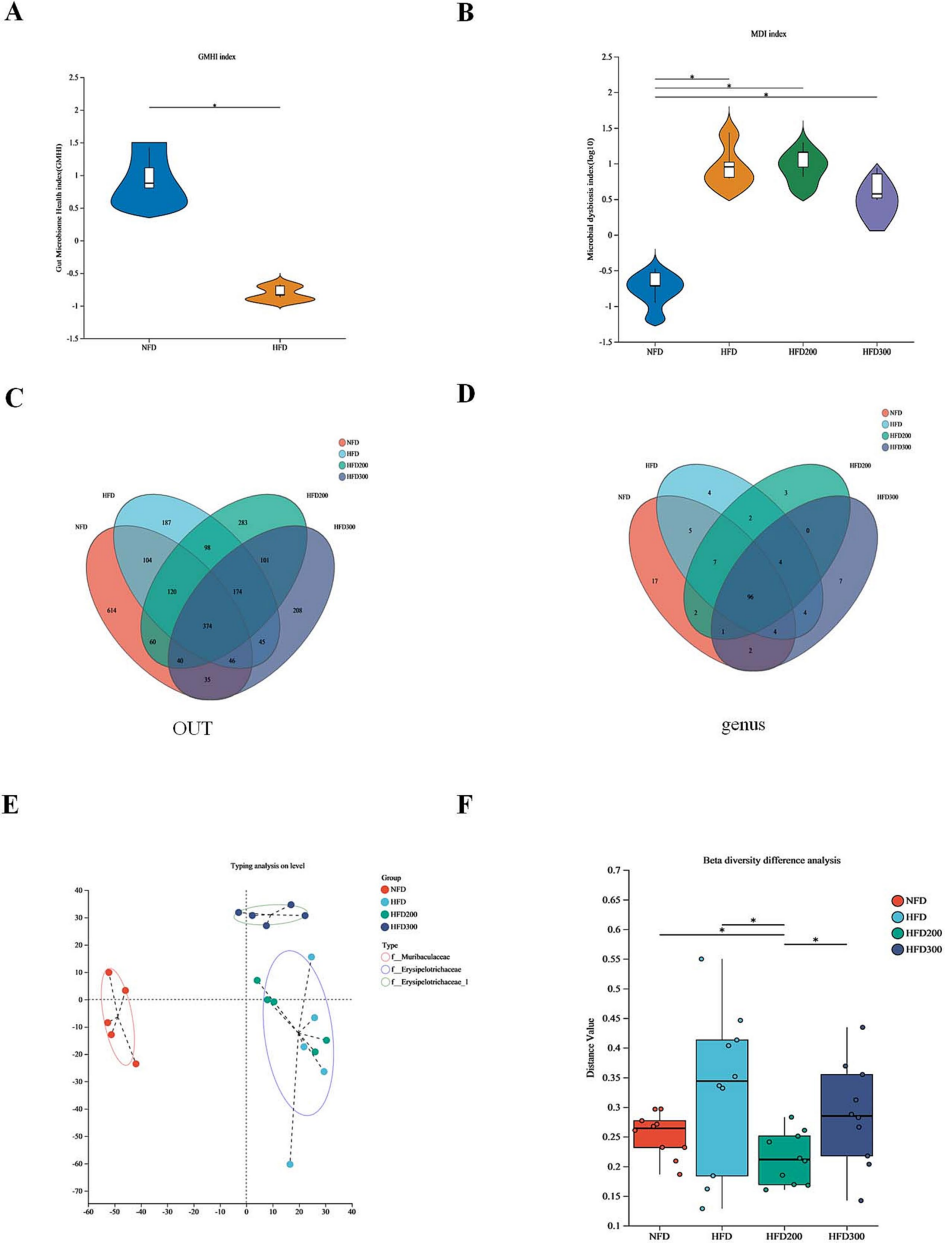
Puerarin intervention alters the structure of gut microbiota. **(A)** The gut microbiome health index (GMHI); **(B)** The microbial dysbiosis index (MDI); **(C)** The Venn diagrams at the OUT level; **(D)** The Venn diagrams at the genus level; **(E)** Typing analysis on level; **(F)** β-diversity difference analysis. *means *p* < 0.05.

Gut microbiota disorder is close to the metabolic dysfunction caused by obesity (Li et al., 2023). The gut microbiome health index (GMHI) is used to describe the health status (Gupta et al., 2020). Compared to NFD, the HFD group exhibited a diminished GMHI index, indicating that a high-fat diet has a discernible adverse impact on the relative abundance of the gut microbiota (Figure 2A). We utilized the microbial dysbiosis index (MDI) to quantify the extent of microbial ecological imbalance (Gunathilake et al., 2020). Our findings indicate that puerarin mitigates gut microbiota disruption induced by HFD (Figure 2B). The Venn diagrams at the OUT level revealed that 374 common OTUs were shared among the groups (Figure 2C). On the OTU level, 96 common genera were identified across all groups. Specifically, the NFD group exhibited 17 unique genera, the HFD group displayed 4 distinct genera, the HFD200 group contained 3 genera, and the HFD300 group had 7 separate genera (Figure 2D). β-diversity analysis, utilizing cluster analysis based on Bray-Curtis distance, segregated four distinct groups of mouse gut microbiota: *f_Muricapillaceae*, *f_ Erysipelotrichaceae*, and *Erysipelotrichaceae_1* (Figures 2E,F). These results suggested puerarin treatment alters the structure of gut microbiota.

### 3.3 Puerarin treatment affects metabolism by altering the composition of gut microbiota

**Figure 3:**
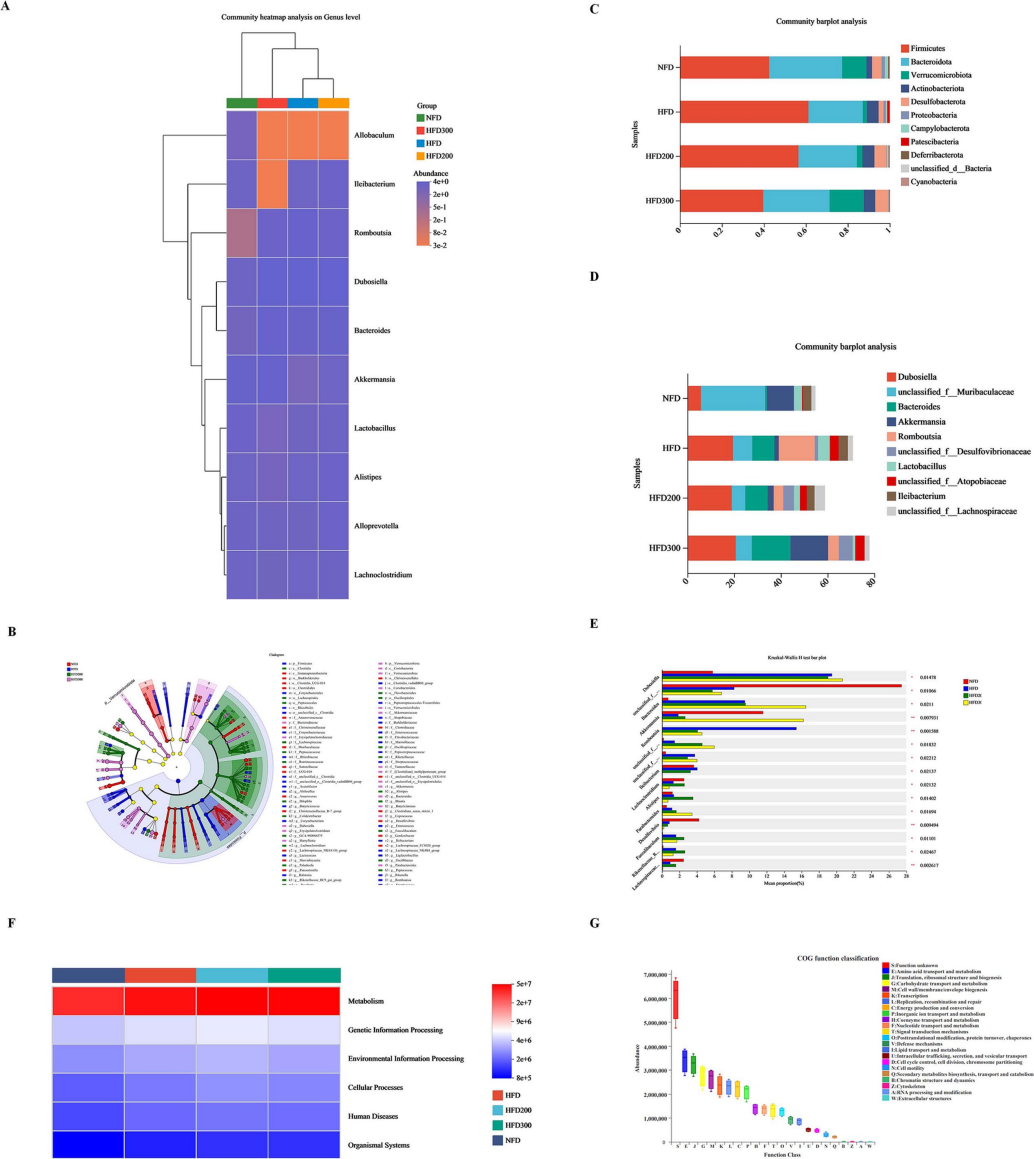
Puerarin treatment affects metabolism by altering the composition of gut microbiota. **(A)** Community heatmap analysis on genus level; **(B)** Lefse multilevel species differentiation discriminant analysis; **(C)** Community barplot analysis at the phylum level; **(D)** Community barplot analysis at the genus level; **(E)** Kruskal-Wallis H test at the genus level; **(F)** PICRUSt2 prediction analysis of the biological functions of the microbiota; **(G)** COG function classification.

To assess the impact of puerarin intervention on gut microbiota composition, we analyzed the microbial communities across all groups at both phylum and genus levels. At the phylum level, we observed an increase in *Firmicutes* in the HFD group, comprising about 61.25%, while *Bacteroidota* and *Verrucomicrobota* proportions decreased to approximately 25.86 and 1.86%, respectively. Puerarin administration reduced *Firmicutes* proportion in a dose-dependent manner (56.50% in the HFD200 group and 39.80% in the HFD300 group), while increasing *Bacteroidota* (27.85% in the HFD200 group and 31.52% in the HFD300 group) and *Verrucomicrobota* (2.65% in the HFD200 group and 16.20% in the HFD300 group) proportions (Figure 3C). At the genus level, *Dunosiella* and *Bacteroides* proportions rose in the HFD group, reaching approximately 19.46 and 9.49%, respectively, while *f__Muribaculaceae* proportions dropped to around 8.26%. Puerarin administration reduced *f__Muribaculaceae* proportions (5.76% in the HFD200 group and 6.83% in the HFD300 group) and increased *Bacteroides* proportions (9.56% in the HFD200 group and 16.47% in the HFD300 group) (Figure 3D). Sequencing data also indicated an increased abundance of *Lleibacterium* following puerarin intervention (Figures 3A,B,E). Based on KEGG database, we conducted a PICRUSt2 prediction analysis of the biological functions of the microbiota. We found that the primary functions of the predicted microbiota are predominantly associated with metabolic processes, especially encompassing amino acid metabolism, transport mechanisms, carbohydrate metabolism, and energy metabolism (Figures 3F,G).

### 3.4 Puerarin intervention reduces the promotion of lipid deposition in adipose tissue

**Figure 4:**
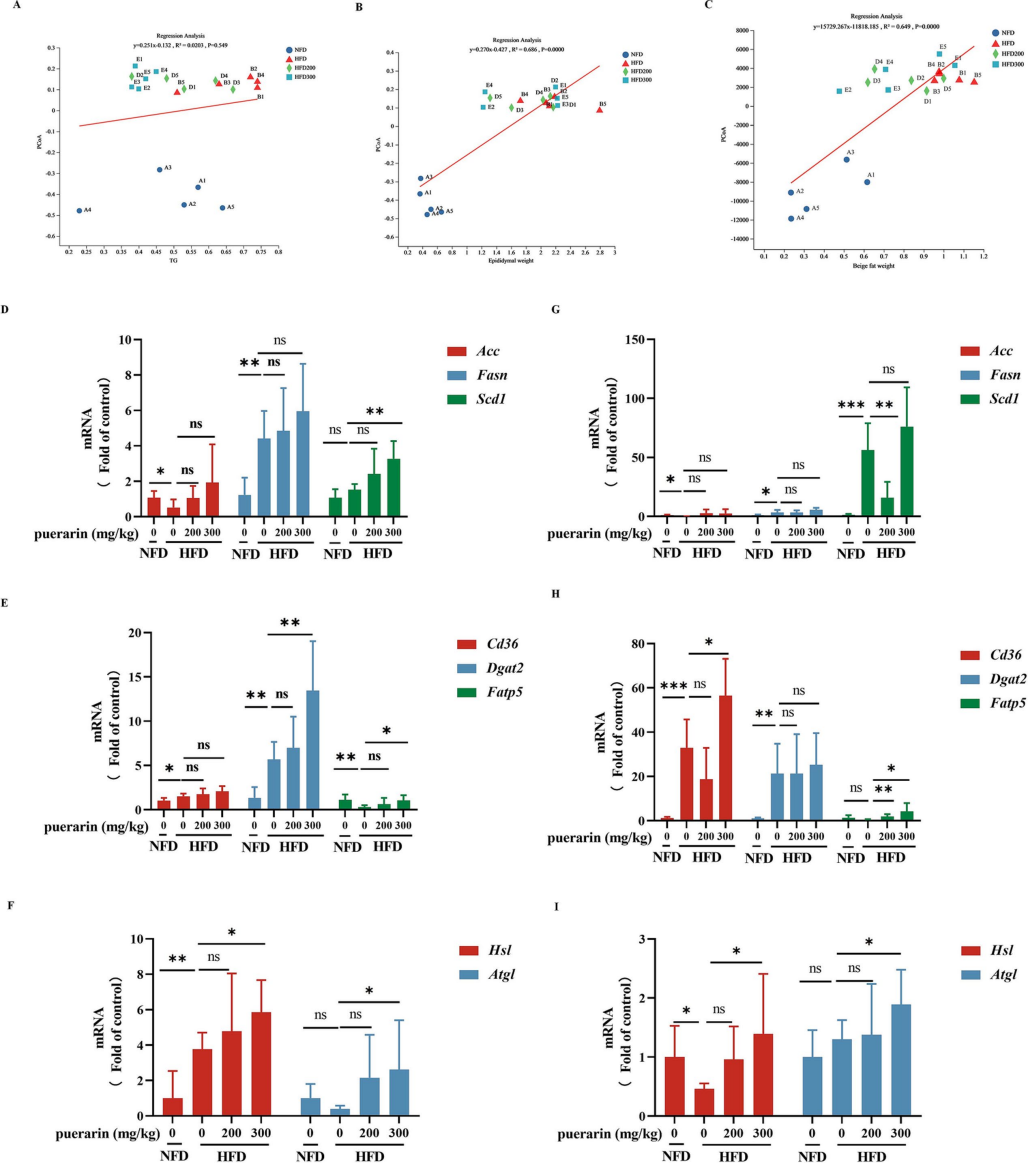
Puerarin intervention reduces the promotion of lipid deposition in adipose tissue. **(A)** Regression analysis between TG and β-diversity; **(B)** Regression analysis between epididymal fat and β-diversity; **(C)** Regression analysis between beige fat and β-diversity; **(D)** qRT-PCR detected the expressions of lipid synthesis (*Acc1, Fasn, Scd1*) in epididymal fat; **(E)** qRT-PCR detected the expressions of fatty acid transport (*Cd36, Dgat2, Fatp5*) in epididymal fat; **(F)** qRT-PCR detected the expressions of triglyceride hydrolysis (*Hsl, Atgl*) in epididymal fat; **(G)** qRT-PCR detected the expressions of lipid synthesis (*Acc1, Fasn, Scd1*) in beige fat; **(H)** qRT-PCR detected the expressions of fatty acid transport (*Cd36, Dgat2, Fatp5*) in beige fat; **(I)** qRT-PCR detected the expressions of triglyceride hydrolysis (*Hsl, Atgl*) in beige fat. *means *p* < 0.05, **means *p* < 0.01, ***means *p* < 0.001.

Linear regression analysis indicates that β-diversity in gut microbiota is not correlated with TG but is associated with variations in the weight of beige and white adipocytes (Figures 4A–C). Lipid deposition in adipose tissue is a prominent feature of obesity. Epididymal fat, classified as a form of white adipose tissue, primarily serves to sequester surplus energy in triglycerides. Its presence holds significant implications for assessing cardiovascular and chronic liver disease risk (Koenen et al., 2021). To evaluate the effects of puerarin by gut microbiota on the regulation of lipid metabolism, we measured the gene expression of lipid synthesis (*Acc, Fasn, Scd1*) (Figure 4D), fatty acid transport (*Cd36, Dgat2, Fatp5*) (Figure 4E), and triglyceride hydrolysis (*Hsl, Atgl*) (Figure 4F) in white adipose tissue. High-dose puerarin increases the gene expression of fatty acid transport (*Dgat2, Fatp5*) and triglyceride hydrolysis (*Hsl, Atgl*) in white adipose. Nevertheless, the administration of puerarin exhibits a minimal impact on lipid synthesis gene expression compared to the HFD group. Beige adipocytes, characterized as inducible adipocytes, exhibit a predominantly white adipose tissue phenotype during thermal equilibrium conditions (Koenen et al., 2021). To investigate puerarin’s impact on beige fat, we assessed the gene expression related to lipid synthesis (*Acc, Fasn, Scd1*) (Figure 4G), fatty acid transport (*Cd36, Dgat2, Fatp5*) (Figure 4H), and triglyceride hydrolysis (*Hsl, Atgl*) (Figure 4I) within the beige adipose. Consistent with the results of white adipose tissue, we found that puerarin treatment significantly increases the gene expression of fatty acid transport (*Cd36, Fatp5*) and triglyceride hydrolysis (*Hsl, Atgl*) in beige fat.

## 4 Discussion

The long-term management of chronic diseases such as obesity presents significant challenges, with high rates of relapse (Martins and Pliner, 2005). This condition often leads to complications over time, evolving into a widespread public health concern (Caruso et al., 2023). Recent research has indicated that puerarin exhibits promising anti-obesity properties (Lyu et al., 2024; Noh et al., 2022). Nonetheless, the precise therapeutic targets of puerarin in addressing obesity remain undefined. Our study demonstrates puerarin mitigates HFD-induced obesity and hyperlipidemia through gut microbiota.

The gut microbiota is widely present in the gastrointestinal tract of mice and participates in the digestive and metabolic processes of dietary intake. Research found that enzymes secreted by different gut microbiota can decompose and transform food components, producing functional by-products such as short-chain fatty acids, trimethylamine-N-oxide, indole and its derivatives, which can have beneficial/harmful effects on the body (Krishnan et al., 2018; Brown et al., 2023). Our result shows that at the phylum level, puerarin intervention can significantly reduce the proportion of *Firmicutes* in the feces and increase the proportion of *Bacteroidota* and *Verrucomiclobota*. At the genus level, puerarin intervention can significantly reduce the proportion of *f_Muribaculaceae* and increase *Bacteroides*. Consistent with previous reports, the ratio of *Firmicutes* to *Bacteroides* exhibits a significant correlation with obesity. Research shows that *Bacteroidetes* can convert polysaccharides into short-chain fatty acids, stimulate leptin secretion, enhance lipid excretion, and ameliorate conditions associated with obesity and hyperlipidemia (Cheng et al., 2022). Moreover, *f_Muribaculaceae* and *Bacteroides* play an important role in inflammation linked to obesity (Sun Y. et al., 2024). It may explain previous reports that puerarin can inhibit the expression of pro-inflammatory cytokines (CCL2, CCL4, CCL5) and TNF-a (Noh et al., 2022). Thus, we speculate that puerarin may ameliorate obesity induced inflammation via the gut microbiota. Therefore, our research indicates that puerarin improves obesity by altering the ratio of *f_Muribaculaceae* to *Bacteroides.* Moreover, there is evidence that the gut microbiota can influence lipid metabolism through the gut microbiota dorsal motor nucleus of the vagus nerve (DMV) pathway, which appears to influence lipid metabolism (Lyu et al., 2024). Therefore, we examined the effect of puerarin on mouse adipose tissue.

Obesity often leads to the expansion of adipose tissue. Although it’s unclear if increased fat mass directly causes metabolic diseases related to obesity, there is strong evidence that patients with higher levels of visceral fat face greater risks of severe complications. Mammalian adipose tissue, includes white adipose tissue, brown adipose tissue, and beige adipose tissue and white adipose tissue responsible for storing excess nutrients, mainly triglycerides (Koenen et al., 2021). Human visceral fat is categorized as white adipose tissue. Research shows that continuous expansion of white fat results in enlarged endoplasmic reticulum and the formation of small lipid droplets within adipocyte cytoplasm, eventually leading to cellular necrosis, inflammation, and extracellular matrix deposition and fibrosis (Santillana et al., 2023). Therefore, controlling the growth of white fat is beneficial for reducing chronic inflammation and fibrosis associated with obesity. In mice, epididymal fat is commonly used as a model for visceral adipose tissue studies. In our study, we found that puerarin administration led to a decrease in white fat mass in mice. Correlation analyses indicated that puerarin’s modulation of gut microbiota was linked to changes in white fat mass. Additionally, we observed alterations in lipid metabolism within white adipose tissue, noting that puerarin intervention significantly boosted triglyceride hydrolysis and fatty acid transport. These findings suggest that puerarin may promote lipolysis in white adipose tissue through its effects on gut microbiota (Marcelin et al., 2019). Compared to white adipose tissue, beige adipose tissue contains more small lipid droplets and dense mitochondria. Its primary function typically resembles that of white adipose tissue—energy storage. However, when exposed to stimuli such as cold temperatures or β-adrenergic agonists, thermogenic genes (*Ucp1, Cidea, Pparα, Pgc1α*) become active, promoting browning and thermogenesis (Wang and Seale, 2016). Similar to observations in white adipose tissue, the genes associated with fatty acid transport and lipolysis (*Hsl, Atgl*) in beige adipose tissue were significantly upregulated compared to the HFD group. Interestingly, we found that low-dose puerarin significantly decreased the expression of *Scd1*, whereas high-dose puerarin increased the *Scd1* expression levels. SCD1 plays a crucial role in desaturating the saturated fatty acids (SFAs) and it is the rate-limiting enzyme of lipid metabolism (Sun Q. et al., 2024). The varying regulatory effects of different doses of puerarin on SCD1 suggest the dual nature of puerarin regulation, but further experimental evidence is needed to confirm.

Our study established an obese mouse model by feeding C57BL/6 mice a high-fat diet. We found that puerarin can improve lipid metabolism in adipose tissue by regulating gut microbiota, promoting the lipolysis of white and beige adipose, and improving mouse obesity. However, our research still has limitations. Our research found that puerarin can improve the structure and composition of the gut microbiota in obese mice, but we can only identify the microbiota at the level of most genera, and we cannot determine which bacterial strain plays a key role in the improvement of obesity. Furthermore, the correlation analysis reveals that the microbiota influenced by puerarin impacts lipolysis in mice. However, additional experimental investigation is required to elucidate the mechanism through which this strain enhances fat hydrolysis in mice.

In summary, our research suggests that puerarin may be a potential dietary composition that can improve obesity through the regulation of gut microbiota structure and composition.

## 5 Conclusion

This study demonstrates that puerarin significantly mitigates high-fat diet-induced obesity and hyperlipidemia through the modulation of gut microbiota. Puerarin treatment alters the composition and structure of fecal microbiota, which in turn affects lipid metabolism by promoting adipose tissue lipolysis and decreasing adipose tissue size. These findings highlight the substantial potential of puerarin as a therapeutic agent for obesity.

## Data Availability

The data presented in the study are deposited in the NCBI repository, accession number PRJNA1236927.

## Footnotes

^1^ https://www.majorbio.com/
